# Reduced Sirtuin1 signalling exacerbates diabetic mice hindlimb ischaemia injury and inhibits the protective effect of a liver X receptor agonist

**DOI:** 10.1111/jcmm.15201

**Published:** 2020-04-14

**Authors:** Wensi Fan, Ran Zhang, Dong Han, Zhenhua Jiang, Shuang Li, Jibin Zhang, Yanhua Li, Yabin Wang, Feng Cao

**Affiliations:** ^1^ Department of Cardiology & National Clinical Research Center for Geriatric Diseases 2nd Medical Center Chinese PLA General Hospital Beijing China; ^2^ Department of Cardiology 1st Medical Center Chinese PLA General Hospital Beijing China; ^3^ Department of Cardiology Xijing Hospital Fourth Military Medical University Xian China; ^4^ Department of Cardiology The General Hospital of Western Theater Command (Chengdu Military General Hospital) Chengdu 610083 China

**Keywords:** diabetes mellitus, endothelial cells, Liver X receptor, peripheral arterial disease, SIRT1

## Abstract

Diabetes mellitus causes endothelial dysfunction, which further exacerbates peripheral arterial disease (PAD). Improving endothelial function *via* reducing endothelial oxidative stress (OS) may be a promising therapy for diabetic PAD. Activation of liver X receptor (LXR) inhibits excessive OS and provides protective effects on endothelial cells in diabetic individuals. Therefore, we investigated the effects of LXR agonist treatment on diabetic PAD with a focus on modulating endothelial OS. We used a streptozotocin‐induced diabetes mouse model combined with a hindlimb ischaemia (HLI) injury to mimic diabetic PAD, which was followed by LXR agonist treatment. In our study, the LXR agonist T0901317 protected against HLI injury in diabetic mice by attenuating endothelial OS and stimulating angiogenesis. However, a deficiency in endothelial Sirtuin1 (SIRT1) largely inhibited the therapeutic effects of T0901317. Furthermore, we found that the underlying therapeutic mechanisms of T0901317 were related to SIRT1 and non‐SIRT1 signalling, and the isoform LXR*β* was involved in LXR agonist‐elicited SIRT1 regulation. In conclusion, LXR agonist treatment protected against HLI injury in diabetic mice *via* mitigating endothelial OS and stimulating cellular viability and angiogenesis by LXR*β*, which elicited both SIRT1‐mediated and non‐SIRT1‐mediated signalling pathways. Therefore, LXR agonist treatment may be a promising therapeutic strategy for diabetic PAD.

## 1 INTRODUCTION

Peripheral arterial disease (PAD) multiplies the risk of non‐traumatic amputation and afflicts over 200 million people worldwide.^1^, ^2^ Diabetes mellitus (DM) increases the morbidity and severity of PAD. One of the major reasons for the increases is that the presence of DM blunts endothelial function, leading to insufficient angiogenesis.^3^ The underlying mechanisms of DM‐induced endothelial dysfunction and death have been demonstrated to include excessive oxidative stress (OS), uncoupled endothelial nitric oxide (NO) synthase and apoptosis.^4^, ^5^ Therefore, a therapeutic method focusing on alleviating endothelial OS and apoptosis should be considered.

As a member of the nuclear receptor superfamily, liver X receptors (LXRs) play pivotal roles in cardiovascular disease and diabetic complications. LXR agonists or genetic treatment can protect against atherosclerosis, myocardial ischaemia/reperfusion injury, myocardial hypertrophy and diabetic cardiomyopathy *via* repressing cellular inflammation, apoptosis and OS damage.^6^, ^7^, ^8^, ^9^ In addition, a previous study also showed that LXR agonist treatment inhibits high glucose (HG)‐induced endothelial OS and senescence, with an additional atheroprotective effect in diabetes.^10^ Hence, we hypothesized that LXR agonist treatment might inhibit endothelial OS and apoptosis, further promoting angiogenesis and protecting against diabetic PAD. To examine this hypothesis, we explored a mouse model of hindlimb ischaemia injury (HLI) with streptozotocin (STZ)‐induced DM, followed by treatment with T0901317, a non‐selective LXR agonist used in our previous study,^11^ to characterize the effects of LXR agonist treatment on diabetic PAD with a focus on endothelial OS and apoptosis.

Silent information regulator 1 (Sirtuin1, SIRT1) is an NAD+‐dependent deacetylase that exerts its regulatory effects on both the nucleus and cytoplasm of endothelial cells (ECs).^12^ A previous study revealed that endothelial SIRT1 ablation exacerbated hypoxic injury and impaired angiogenesis.^13^ In contrast, ECs were rescued from hypoxic exposure through SIRT1 up‐regulation.^14^ Significantly, SIRT1 is essential for healthy vasculature, as endothelial SIRT1 deficiency leads to increased OS, inflammation and senescence.^15^ Furthermore, a previous study showed that SIRT1 also deacetylates and activates LXR,^16^ and the SIRT1‐LXR axis contributes to atheroprotection by reducing inflammation.^17^ Interestingly, our previous research demonstrated that LXR agonist treatment activated SIRT1, deacetylating its downstream signals and protecting myocardial cells *via* inhibiting OS and apoptosis during sepsis‐induced myocardial injury.^11^ However, the interplay between endothelial SIRT1 and LXR in response to diabetic PAD is still unclear. To elucidate this, we used endothelial‐specific SIRT1 knockout mice treated with T0901317 to investigate the interaction between SIRT1 and LXR and evaluate the potential effects of LXR agonist treatment on diabetic PAD.

## 2 MATERIALS AND METHODS

### 2.1 Experimental animals

To generate endothelial‐specific SIRT1 knockout mice, Tie2‐Cre mice were mated with SIRT1^loxp^ mice. Tie2‐Cre mice that were on a C57BL/6 background were purchased commercially (number: 004 128, Jackson Laboratory); specifically, the mice possessed a Cre recombinase‐oestrogen receptor fusion protein under regulation of endothelial receptor tyrosine kinase (Tie2) promoter. LoxP‐flanked (floxed) SIRT1 allele (SIRT1^loxp^) mice were generously presented by Prof. Yongzhan Nie, as reported in a previous study.^11^ PCR was performed for genotype identification. Male littermates were matched with age and weight (6‐8 weeks, 20‐25 g).

### 2.2 Animal groups and treatment

SIRT1^endo−/−^ mice or their wild‐type littermates were randomly divided into five groups: (1) wild‐type HLI group (HLI group, n = 20), (2) diabetic wild‐type HLI group (HLI + DM group, n = 20), (3) diabetic wild‐type HLI with T0901317 treatment group (HLI + DM+LXR group, n = 20), (4) diabetic endothelial‐specific SIRT1 knockout HLI with T0901317 treatment group (HLI + DM+LXR + SIRT1^endo−/−^ group, n = 20) and (5) diabetic endothelial‐specific SIRT1 knockout HLI group (HLI + DM+SIRT1^endo−/−^ group, n = 20). The diabetes model was induced through intraperitoneal injection of STZ (50 mg/kg) after 12 hours of fasting for 5 successive days. Three months later, mice with a random blood glucose levels (measured by a glucometer; Bayer Corporation) that were greater than 16 mmol/L were considered diabetic. Plasma insulin contents were evaluated using commercial ultra‐high mouse insulin ELISA kits (Antibody and Immunoassay Services) in accordance with the manufacturer's instructions.

Mice in groups (3) and (4) had established HLI and were treated with the LXR agonist T0901317 (30 mg/kg/day; Cayman Chemical) by gavage for 21 consecutive days. Groups (1), (2) and (5) were treated with vehicle (1% ethanol in normal saline) by the same method for the corresponding period.

The HLI model was established as our previous study.^4^ All procedures were performed in accordance with the Guide for the Care and Use of Laboratory Animals publication from the NIH (No. 85‐23, revised 1985 and updated 2011). The protocol was approved by the Fourth Military Medical University ethics review board (XJLL2015065).

### 2.3 Cell culture and treatment

Human umbilical vein endothelial cells (HUVECs) were obtained and cultured using the same method as our previous study.^4^ HUVECs were cultured at a concentration of 5.5 mmol/L glucose for the normal treatment or 33.3 mmol/L, which represented the HG treatment. SIRT1, LXR*α* and LXR*β* small interfering RNAs (siRNAs) were purchased from Santa Cruz and were transfected at a concentration of 100 nmol/L. After the indicated treatment, HUVECs were exposed to hypoxia (95% N_2_ /5% CO_2_)/ serum deprivation (H/SD) treatment for 6 hours.

### 2.4 Serial laser Doppler perfusion imaging of hindlimb

Laser Doppler perfusion imaging (LDPI, PeriScan‐PIM3; Perimed) was performed to evaluate blood perfusion recovery of ischaemic hindlimbs. The perfusion ratio (PR, ratio of average LDPI index of ischaemic to non‐ischaemic) was utilized to qualify the perfusion recovery. From an animal ethics point of view, mice with autoamputation were killed immediately, and PR was defined as 0.0 on postoperative day (POD) 21.

### 2.5 Ischaemic characterization score and ambulatory impairment evaluation

Semiquantitative assessments were performed to evaluate HLI injury and ambulatory impairment. Briefly, the ambulatory impairment score was defined as: 0 = toe flexion, 1 = foot flexion, 2 = no dragging but plantar flexion, 3 = foot dragging or autoamputation. The ischaemic characterization score was described as follows: 0 = no change, 1 = mild discoloration, 2 = severe discoloration, 3 = necrosis, 4 = autoamputation.

### 2.6 Vascular casting mould assay

Gastrocnemius tissue was harvested for casting neovasculature on POD 21. Distal vessels were cast and sputter‐coated with gold. Then, the mould was fixed by conducting resin and photographed using scanning electron microscopy (S‐4800, Hitachi).

### 2.7 Histological staining

Gastrocnemius tissue was fixed with 4% paraformaldehyde and sectioned on POD 7 or POD 21. Morphological changes in gastrocnemius tissues were shown by haematoxylin‐eosin (H*&*E) staining. Immunofluorescent staining was performed to detect the expression of *α*‐SMA and CD31 for assessing angiogenesis. Slices were incubated with primary antibodies against *α*‐SMA (Abcam, ab32575) and CD31 (Abcam, ab24590); then, they were stained with the respective fluorescent secondary antibodies. CD31 was co‐stained with SOD2 (Abcam, ab13533) or was stained together with terminal deoxynucleotidyl transferase‐mediated dUTP nick end labelling (TUNEL, Sigma‐Aldrich) to evaluate endothelial antioxidant levels and apoptosis. Immunofluorescent pictures were captured under a laser confocal microscope (FluoView‐FV1000, Olympus).

### 2.8 Measurement of reactive oxygen species

The levels of reactive oxygen species (ROS) in tissue and in cells were detected by the dihydroethidium (DHE, Beyotime, S0063) as described in a previous study.^4^ Gastrocnemius tissue or HUVECs were labelled with a DHE probe (5 μmol/L) for 20 minutes in a dark incubator at 37℃. Fluorescence microscope was used to observe sections at 535 nm excitation.

Moreover, to detect the mean fluorescence density (autofluorescence modification), cells (1x10^5^) were resuspended and labelled with DHE (5 μmol/L). Flow cytometry (Becton Dickinson Biosciences) was used to measure the fluorescence.

### 2.9 Measurement of intracellular NOx

Intracellular NOx production (NO and its oxidized forms) was detected using a commercial nitrate/nitrite assay kit (Beyotime, S00233). The indicated samples were prepared in accordance with the manufacturer's instructions. A luminometer was utilized to detect the absorbance at 540 nm. NOx contents were expressed as nmol/10^5^ cultured cells.

### 2.10 Detection of cytokines and OS‐related indicators

Gastrocnemius tissue was collected and homogenized to evaluate angiogenesis and OS‐related indicators. The expression of vascular endothelial growth factor (VEGF) and basic fibroblast growth factor (bFGF) was evaluated using commercial ELISA kits (R*&*D Systems) in accordance with the manufacturer's instructions. OS‐related indicators: glutathione (GSH), malondialdehyde (MDA), catalase (CAT) and superoxide dismutase (SOD) in tissues were evaluated using the commercial testing kits, respectively (all purchased from Beyotime), following the manufacturer's instructions. MDA and GSH were expressed as contents (nm/mg protein), and SOD and CAT were expressed as enzymatic activity units (U/mg protein). A microplate reader (Thermo Scientific) was used to measure the results.

### 2.11 Tube formation assay

Matrigel (BD Biosciences) was pre‐cooled and placed in 96‐well dishes for 40 minutes. HUVECs were plated in each well at a concentration of 3 × 10^4^/100 μL. Tubes were observed with an inverted phase contrast microscope (Nikon) after 6 hours of incubation. Tube lengths were calculated with ImageJ software using an angiogenesis analyser (ImageJ News. 2012).

### 2.12 Cell migration assay

HUVECs were resuspended at a concentration of 5 × 10^4^/100 μL and then placed in a migration chamber, which was immersed in a 24‐well dish filled with 500 μL of medium. After 24‐hours incubation, migrated cells were fixed with 4% paraformaldehyde and dyed with 0.1% crystal violet. Positive cells were counted in four random fields for each well.

### 2.13 Caspase‐3 activity assay

Gastrocnemius tissue was homogenized to assess caspase‐3 activity using a caspase‐3 activity kit (Beyotime, C1116), according to the manufacturer's instruction. The actual OD405 was calculated by subtracting the blank control without pNA, and the results are presented as μM/h/g.

### 2.14 Real‐time quantitative PCR

HUVECs were lysed using TRIzol reagent to extract total RNA. Reverse transcription was performed using an Omniscript RT Kit (Qiagen), followed by amplification of the cDNA with Fast SYBR Green Master Mix (TAKARA Biotechnology) on an ABI 7900HT System. The sequences of the forward and reverse primers were as follows: SIRT1 (forward) 5′‐GCCAGAGTCCAAGTTTAGAAGA‐3′, (reverse) 5′‐CCATCAGTCCCAAATCCAG‐3′; GAPDH (forward) 5′‐TGGAAGGACTCATGA CCACA‐3′, (reverse): 5′‐TTCAGCTCAGGGATGACCTT‐3′. The relative SIRT1 mRNA transcript levels were calculated by normalizing them to GAPDH and expressed the data as a relative ratio.

### 2.15 Western blotting

Gastrocnemius tissue and HUVECs were lysed to extract proteins. Protein lysates (50 μg) were loaded and separated on the SDS‐PAGE gel. Then, proteins were transferred onto nitrocellulose membranes. Membranes were incubated with the corresponding primary antibodies against cleaved caspase‐3 (Abcam, ab49822), caspase‐3 (Abcam, ab13847), Bcl‐2 (Abcam, ab182858), BAX (Abcam, ab32503), LXR*α* (Abcam, ab176323), LXR*β* (Abcam, ab28479), NOX4 (Abcam, ab109225), 3‐Nitrotyrosine (Abcam, ab52309), SIRT1 (Cell Signaling, #9475), hydroxylated HIF‐1*α* (Cell Signaling, #3434), FoxO1 (Cell Signaling, #2880), acetyl‐FoxO1 (Santa Cruz, sc‐49437), p53 (Cell Signaling, #2524), acetyl‐p53 (Cell Signaling, #2570) and *β*‐actin (Abcam, ab8227). Membranes were treated with an enhanced chemiluminescence kit (Millipore) and were imaged with UVP Bio‐Imaging Systems. QuantiOne imaging software (Bio‐Rad) was used for quantitative analysis by evaluating the integrated optical density.

### 2.16 Statistical analysis

Results were presented as the mean ± SEM. Prism 6.0 (GraphPad Software) was used for analyses. Two groups were compared utilizing Student's two‐tailed unpaired t test. One‐way factor analysis of variance analysis was used for multi‐group comparisons, followed by Dunnett's post hoc test. *P* values < .05 were regarded as statistically significant.

## 3 RESULTS

### 3.1 Endothelial‐specific SIRT1 deletion exacerbated hindlimb ischaemia injury in diabetic mice and inhibited the LXR‐mediated protective effects

**Figure 1:**
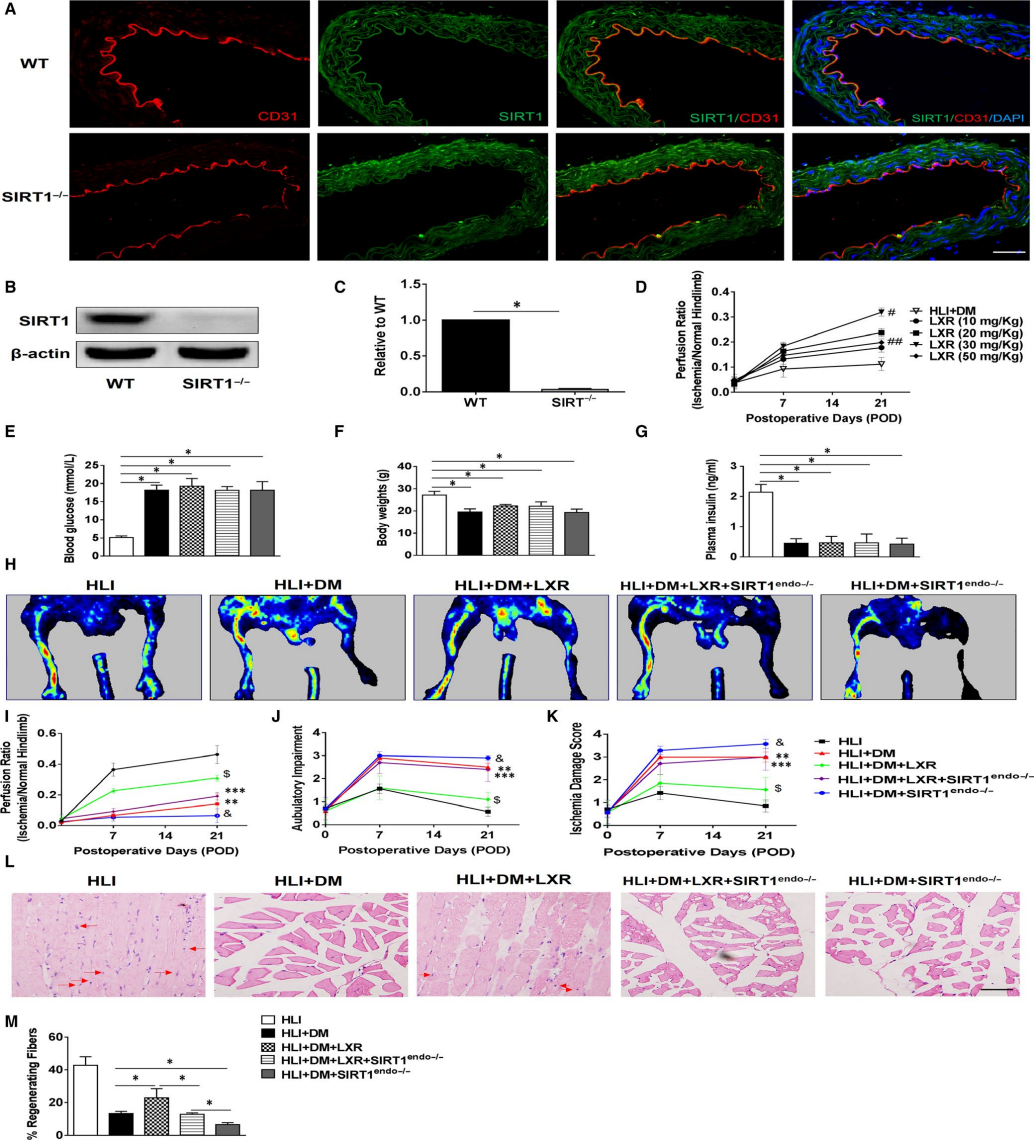
Effects of the LXR agonist T0901317 on hindlimb ischaemia injury in diabetic mice. A, Visualization of endothelial‐specific SIRT1 deletion [CD31 (endothelial tissue, red fluorescence) and SIRT1 (green fluorescence)]. Scale bar: 50 µm. B‐C, Western blotting was used to analyse endothelial SIRT1 expression (n = 5). D, Therapeutic effects of different doses of the LXR agonist T0901317 on diabetic HLI injury (n = 6). E‐G, Random blood glucose, bodyweight and plasma insulin. H, Laser Doppler perfusion imaging (LDPI) visualized dynamic changes in hindlimb perfusion on POD 21. I, Blood perfusion was quantified using the perfusion ratio (PR, the average LDPI of ischaemic/non‐ischaemic hindlimb). n = 10. J, Scores of ambulatory impairment. n = 6‐10 for each group. K, Scores of ischaemia damage. n = 6‐10 for each group. L, Representative images of regenerated gastrocnemius (defined as nucleus centralization and intensive arrangement, red arrows indicated) by H&E staining on POD 21. Scale bar: 100 µm. M, Quantification of the percentage of regenerating fibres, which are characterized by the presence of a centrally located nucleus. (n = 5). Error bars represent the mean ± SEM. *P < .05 between the indicated groups. *#P* < .05 between HLI + DM and HLI + DM+LXR (30 mg/kg). ##*P* < .05 between HLI + DM+LXR (30 mg/kg) and HLI + DM+LXR (50 mg/kg). **P < .05 between HLI + DM and HLI + DM+SIRT1^endo−/−^. ***P < .05 between HLI + DM+LXR and HLI + DM LXR + SIRT1^endo−/−^. $*P* < .05 between HLI + DM+LXR and HLI + DM. &*P* < .05 between HLI + DM and HLI + DM+SIRT1^endo−/−^

First, the aorta ventralis of Tie2‐Cre‐SIRT1^Loxp+/+^ mouse was sectioned to evaluate endothelial SIRT1 expression through immunofluorescent staining. As shown in Figure 1A, endothelial SIRT1 was deleted specifically, as evidenced by the presence of only CD31 red fluorescence. Furthermore, mouse aortic ECs were isolated through repeating a differential adhesion procedure and were cultured in accordance with a previous report.^18^ Endothelial SIRT1 expression was detected by Western blotting, and the results are shown in Figure 1B‐C. The expression of endothelial SIRT1 decreased markedly in Tie2‐Cre‐SIRT1^Loxp+/+^ mice compared with that of wild‐type mice.

To establish the dose of the LXR agonist T0901317 for the following study, diabetic mice were treated with T0901317 at doses of 10 mg, 20 mg, 30 mg and 50 mg/kg for 21 days after HLI injury. As shown in Figure 1D, PR was restored with increasing doses of T0901317. The 30 mg/kg dose had a better therapeutic effect than any other dose; this effect was weakened at a dose of 50 mg/kg. Therefore, a dose of 30 mg/kg was used in our subsequent study.

Three months after STZ injection, mouse blood glucose levels increased significantly compared to that of normal mice (Figure 1E). T0901317 did not decrease the levels of blood glucose, with or without endothelial‐specific SIRT1 deletion. STZ injection also dramatically reduced mouse body weight and plasma insulin content, with or without endothelial‐specific SIRT1 deletion (Figure 1F‐G).

Endothelial‐specific SIRT1 deletion inhibited hindlimb blood reperfusion in mice with diabetic HLI injury compared with that of HLI + DM mice, as evidenced by decreased PR in the HLI + DM+SIRT1^endo−/−^ group (Figure 1H‐I). T0901317 treatment recovered hindlimb blood perfusion compared with that of HLI + DM mice; however, this beneficial effect was largely weakened in SIRT1^endo−/−^ mice (Figure 1H‐I). Interestingly, LXR agonist treatment partially restored blood perfusion in SIRT1^endo−/−^ mice compared to that of the HLI + DM+SIRT1^endo−/−^ group (Figure 1H‐I).

As shown in Figure 1J‐K, endothelial‐specific SIRT1 deletion exacerbated ischaemic symptoms and greatly impaired ambulatory ability compared to those of HLI + DM mice. T0901317 treatment facilitated diabetic mice hindlimb functional recovery and ameliorated ischaemic symptoms compared to those of the HLI + DM group; however, these effects were inhibited in the SIRT1^endo−/−^ mice (Figure 1J‐K). Consistent with the PR results, LXR agonist treatment partially restored hindlimb function in SIRT1^endo−/−^ mice compared to that of HLI + DM+SIRT1^endo−/−^ mice (Figure 1J‐K). Furthermore, histological results were consistent with semiquantitative assessments, as evidenced by regenerated and functional myofibres (indicated by the red arrows), which were detected by haematoxylin‐eosin staining (Figure 1L‐M).

### 3.2 Endothelial‐specific SIRT1 deletion restrained ischaemia‐induced angiogenesis in diabetic mice and inhibited the pro‐angiogenetic effect of LXR agonist treatment

**Figure 2:**
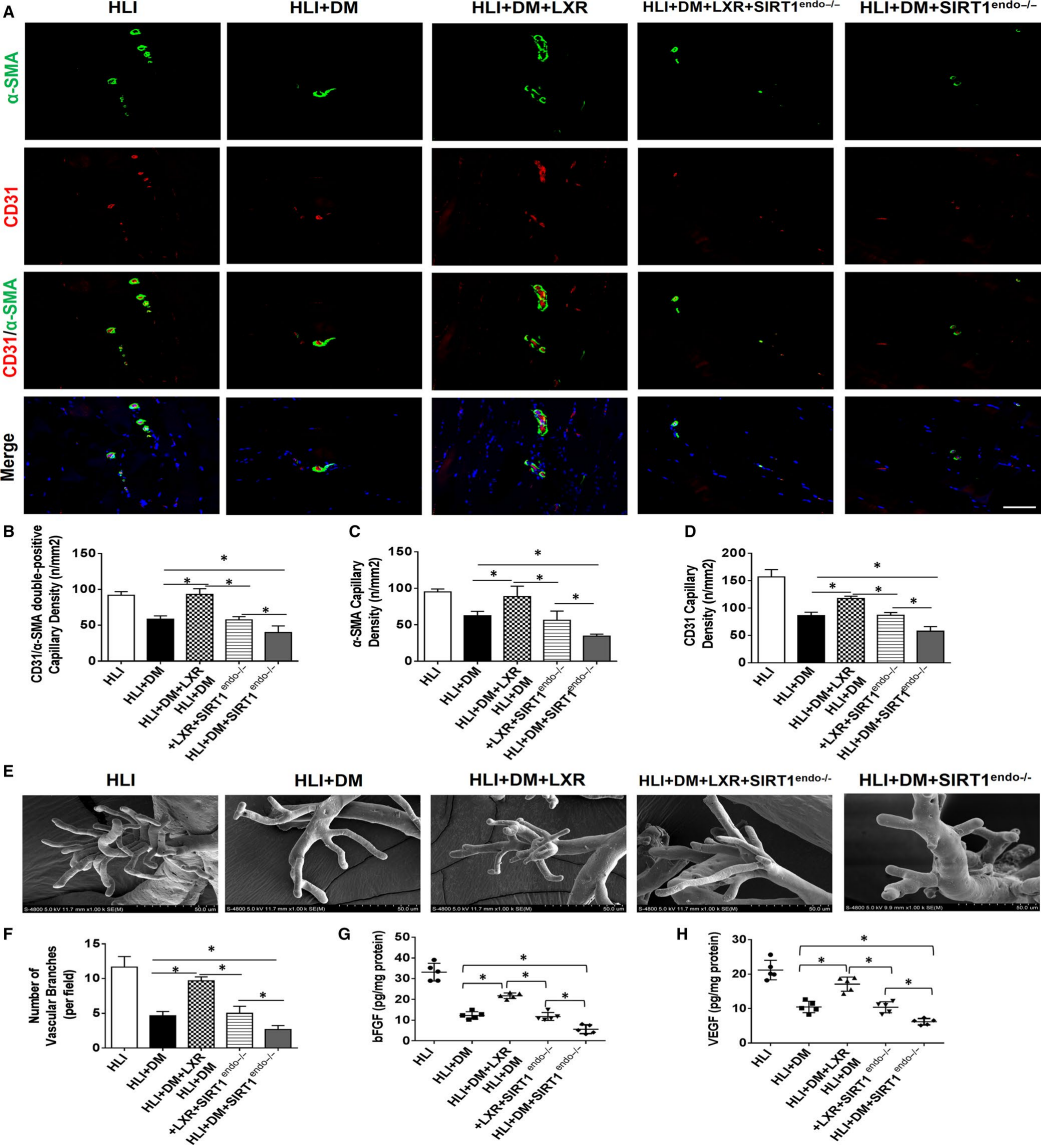
The LXR agonist T0901317 promoted ischaemic hindlimb angiogenesis in diabetic mice. A, Representative images of mature blood vessels [consisting of endothelial cells (CD31 red fluorescence) covered by smooth muscle cells (*α*‐SM‐actin green fluorescence)]. Scale bar: 50 µm. B, Quantitative analysis of CD31 and *α*‐SM‐actin double‐positive capillary density (n = 5). C, Quantitative analysis of *α*‐SM‐actin capillary density (n = 5). D, Quantitative analysis of CD31 capillary density (n = 5). E, Hindlimb vascular casting mould images were captured by scanning electron microscopy to evaluate new vessels on POD 21. F, Quantitative analysis of vascular branches (n = 5). G and H, bFGF and VEGF content in gastrocnemius tissue were measured by ELISA on POD 21 (n = 5). *P < .05 between the indicated groups

*α*‐SMA and CD31 co‐staining were performed to evaluate angiogenesis and to determine the density of mature blood vessels. Endothelial‐specific SIRT1 deletion further inhibited ischaemia‐induced angiogenesis in diabetic mice, as evidenced by the decreased density of *α*‐SMA, CD31 and *α*‐SMA/CD31 in the HLI + DM+SIRT1^endo−/−^ group (Figure 2A‐D). LXR agonist treatment stimulated ischaemic hindlimb angiogenesis and increased the density of mature blood vessels compared to those of HLI + DM mice; however, these effects were abrogated by the endothelial‐specific SIRT1 deletion (Figure 2A‐D). However, LXR agonist treatment still increased the density of *α*‐SMA, CD31 and *α*‐SMA/CD31 by a small amount in SIRT1^endo−/−^ mice compared to that of the HLI + DM+SIRT1^endo−/−^ group (Figure 2A‐D). Furthermore, vascular casting mould assay further confirmed the results of the histological staining (Figure 2E‐F). Additionally, the levels of pro‐angiogenetic factors (bFGF and VEGF) in the gastrocnemius were consistent with the histological results (Figure 2G‐H).

### 3.3 Endothelial‐specific SIRT1 deletion exacerbated ischaemia‐induced endothelial apoptosis in diabetic mice and weakened the LXR‐mediated anti‐apoptotic effect

**Figure 3:**
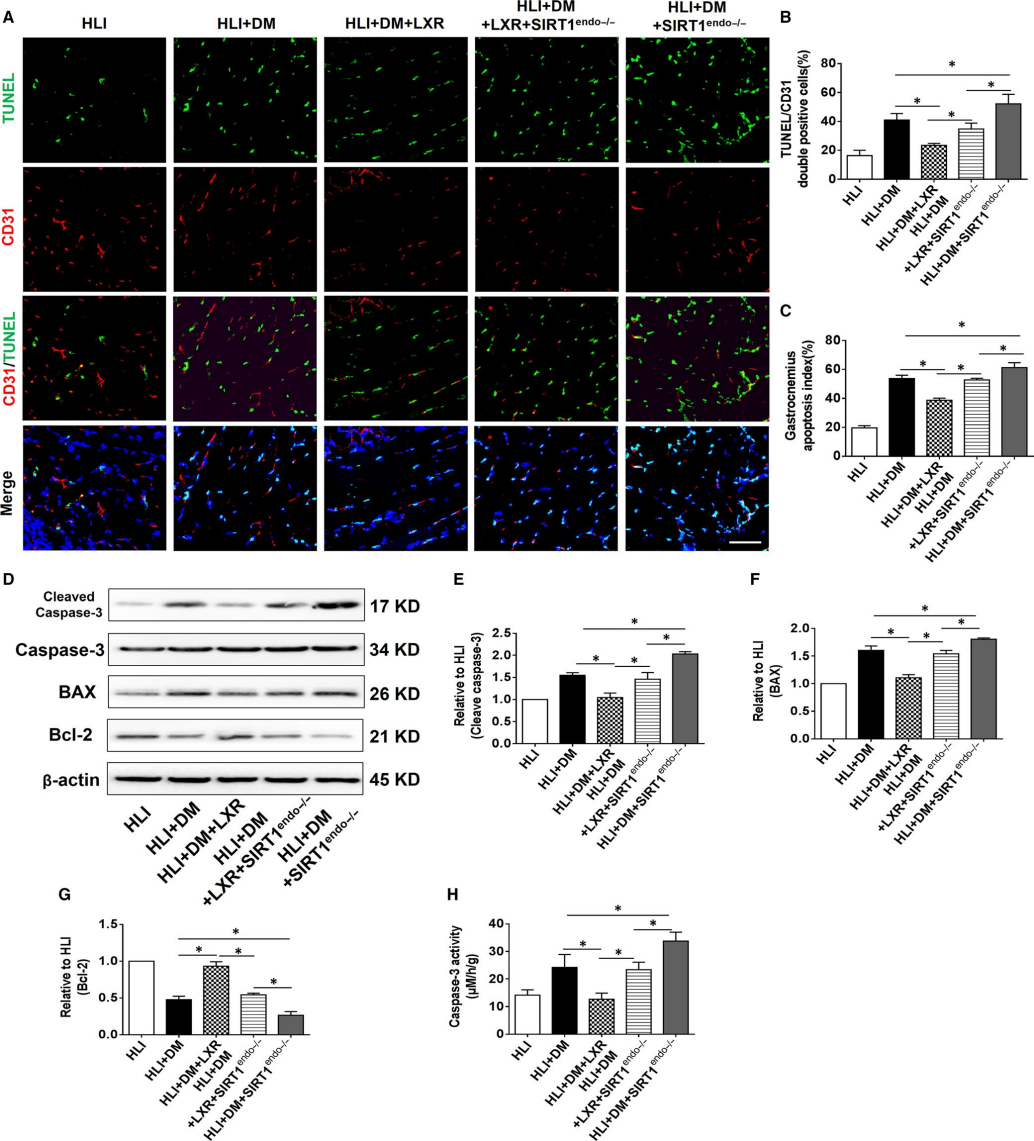
The LXR agonist T0901317 attenuated endothelial and gastrocnemius apoptosis in diabetic HLI mice. A, Endothelial cells and gastrocnemius apoptosis were detected with TUNEL assay (apoptotic cells, green fluorescence), CD31 staining (endothelial cells, red fluorescence) and DAPI staining (total nuclei, blue fluorescence) on POD 7. Scale bar: 50 µm. B, Endothelial apoptosis index (green and red double‐positive/ total nucleus, n = 5). C, Gastrocnemius apoptosis index (total TUNEL positive/ total nucleus, n = 5). D, Representative blots of cleaved caspase‐3, caspase‐3, BAX, and Bcl‐2 (E‐G) Western blotting analysed the expression of cleaved caspase‐3, BAX, and Bcl‐2 in the gastrocnemius on POD 7 (n = 5). H, Caspase‐3 activity assays were performed to evaluate gastrocnemius apoptosis (n = 5). *P < .05 between the indicated groups

CD31 staining and TUNEL assays were performed to evaluate endothelial apoptosis, which was characterized as double‐positive staining of POD 7. As shown in Figure 3A‐C, endothelial‐specific SIRT1 deletion exacerbated ischaemia‐induced endothelial and gastrocnemius apoptosis in diabetic mice compared to that of HLI + DM mice. LXR agonist treatment attenuated endothelial and gastrocnemius apoptosis; however, it did not attenuate apoptosis in SIRT1^endo−/−^ mice compared with that in the HLI + DM+LXR group (Figure 3A‐C). However, LXR agonist treatment still resulted in an anti‐apoptotic effect in SIRT1^endo−/−^ mice compared to that in the HLI + DM+SIRT1^endo−/−^ group (Figure 3A‐C). Furthermore, the levels of cleaved caspase‐3 and BAX or the anti‐apoptotic protein Bcl‐2 were consistent with the results of gastrocnemius histological staining (Figure 3D‐G). The caspase‐3 activity assay further confirmed the Western blotting results (Figure 3H).

### 3.4 Endothelial‐specific SIRT1 deletion exacerbated ischaemia‐induced endothelial cell OS in diabetic mice and weakened the LXR‐mediated antioxidative effect

**Figure 4:**
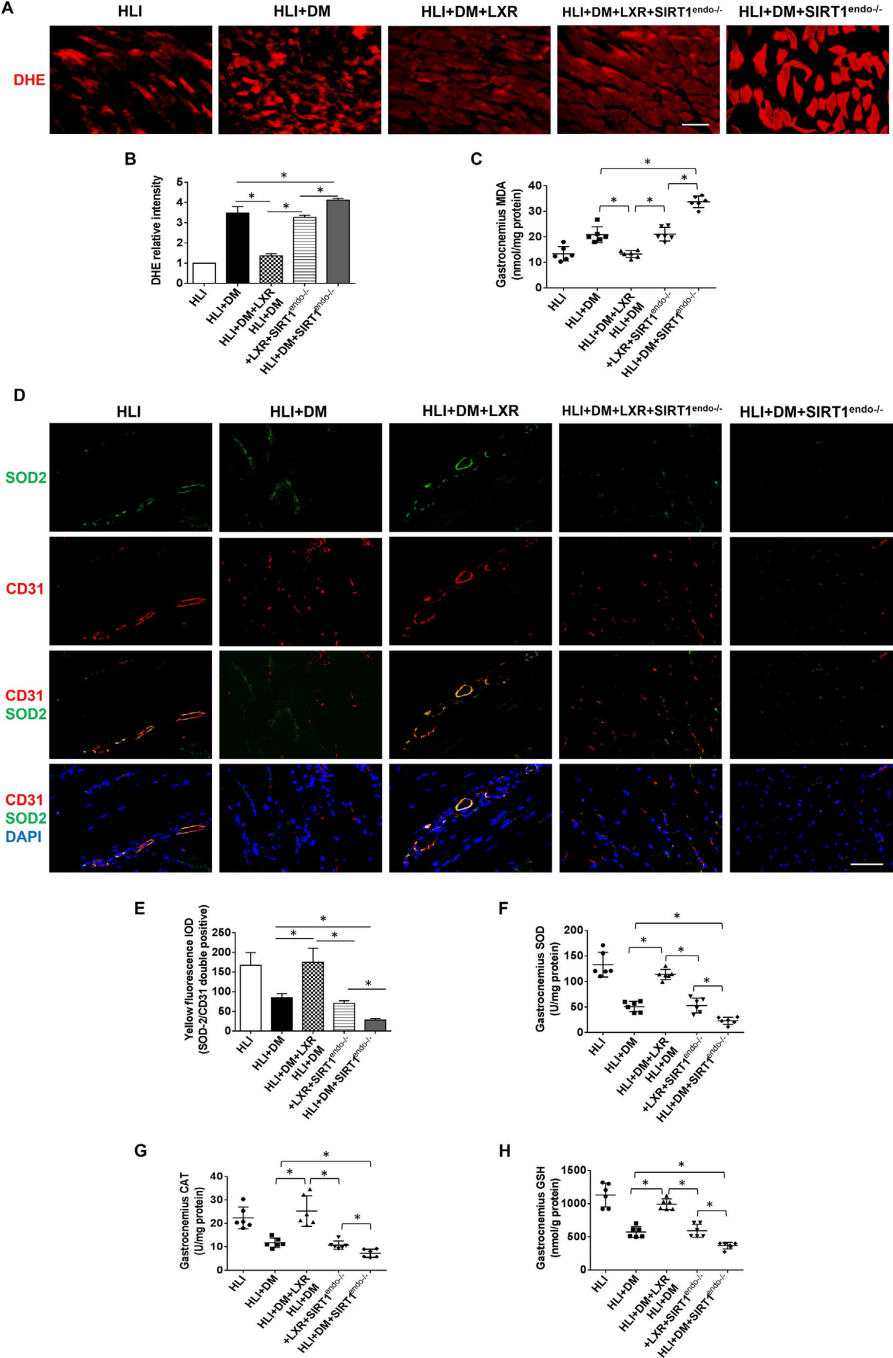
The LXR agonist T0901317 mitigated ischaemic hindlimb oxidative stress and promoted endothelial antioxidant machinery in diabetic mice. A, Representative images are shown of dihydroethidium fluorescence (red) staining which detects ROS generation in the left gastrocnemius on POD 7. Scale bar: 50 µm. B, Quantitative analysis of DHE relative intensity (n = 5). C, Measurement of the oxidative stress‐related indicator malondialdehyde (MDA) in gastrocnemius tissues (n = 6 for each group). D, Representative immunofluorescent staining for endothelial SOD‐2 expression [CD31 and SOD‐2 double‐positive (yellow fluorescence) and DAPI (blue fluorescence)] on POD 7. Scale bar: 50 µm. E, Quantitative analysis of yellow fluorescence IOD (CD31 and SOD‐2 double‐positive) (n = 5). F‐H, Measurement of antioxidant superoxide dismutase (SOD), catalase (CAT), and glutathione (GSH) in gastrocnemius tissues (n = 6 for each group). *P < .05 between the indicated groups

As shown in Figure 4A‐B, endothelial‐specific SIRT1 deletion exacerbated gastrocnemius OS compared to that of the HLI + DM group, as evidenced by the increase in DHE intensity. LXR agonist treatment mitigated ischaemia‐induced OS in diabetic mice; however, this effect was counteracted by endothelial deletion of SIRT1 (Figure 4A‐B). The trend in gastrocnemius MDA levels was consistent with DHE intensity (Figure 4C), further confirming the results of DHE staining.

Furthermore, CD31 and SOD‐2 were co‐stained to evaluate endothelial antioxidative stress factors, which were characterized as double‐positive fluorescence (yellow). Endothelial‐specific SIRT1 deletion decreased the intensity of yellow fluorescence in the HLI + DM group compared to that in the HLI + DM group (Figure 4D‐E). LXR agonist treatment increased the expression of endothelial SOD‐2 in HLI + DM mice; however, similar results were not observed in HLI + DM+SIRT1^endo−/−^ mice. Furthermore, we also found that the levels of endogenous antioxidants (SOD, CAT and GSH) in the gastrocnemius increased notably in the HLI + DM+LXR group (*vs* HLI + DM group); however, similar results were not observed in the HLI + DM+LXR + SIRT1^endo−/−^ group (*vs* HLI + DM+LXR group). Moreover, in the group with the endothelial‐specific SIRT1 deletion, LXR agonist treatment also inhibited OS in HLI + DM+SIRT1^endo−/−^ mice compared to that of the untreated HLI + DM+SIRT1^endo−/−^ group (Figure 4F‐H).

### 3.5 SIRT1 silencing increased apoptosis, decreased tube formation and migration of HUVECs in response to H/SD + HG, and it weakened the protective effects of T0901317

**Figure 5:**
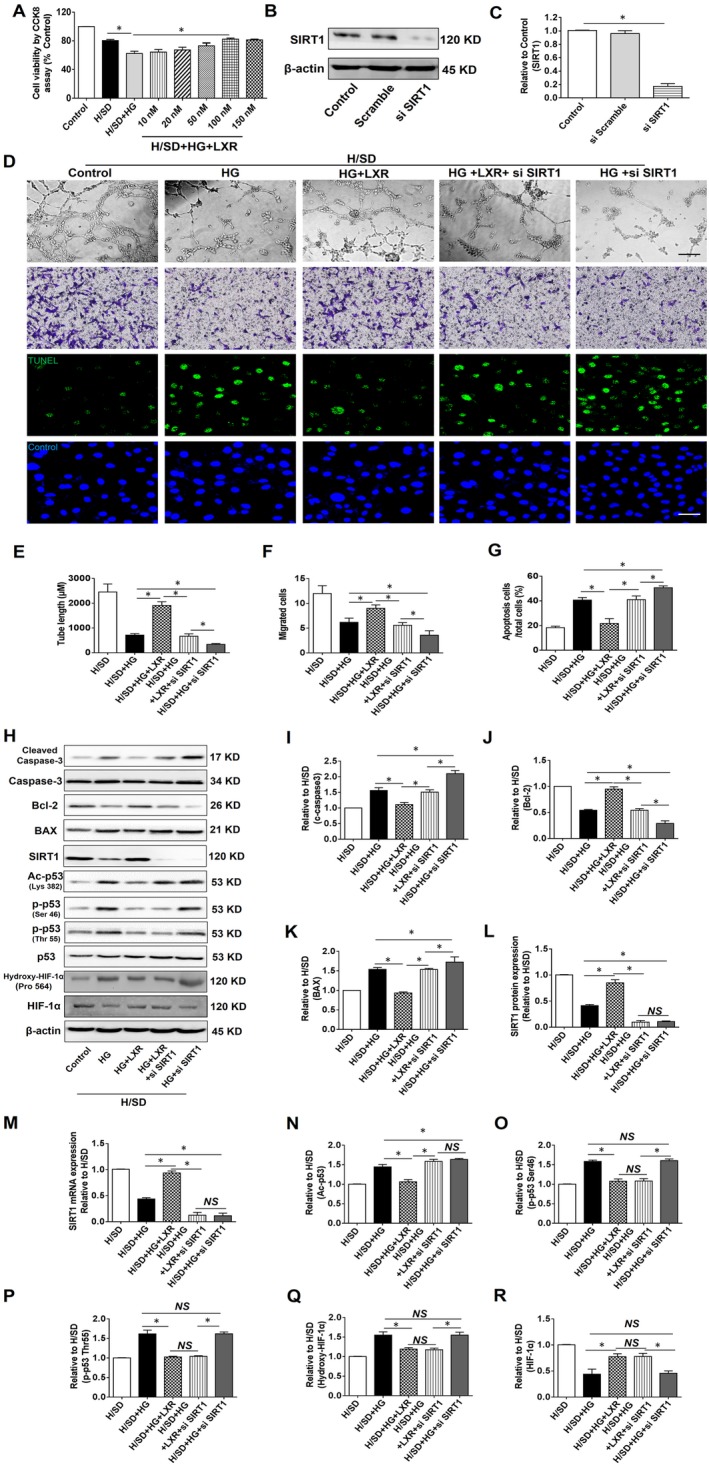
The LXR agonist T0901317 inhibited apoptosis and increased tube formation and migration of HUVECs in H/SD + HG injury. A, Effects of different concentrations of the LXR agonist T0901317 on HUVEC viability in H/SD + HG injury. n = 5. B‐C, The effects of SIRT1 siRNA intervention were assessed by Western blotting (n = 3). D, Representative images of tube formation and migration, scale bar: 100 µm. Representative images of TUNEL assay for assessing apoptosis, scale bar: 30 µm. E, Quantitative analysis of tube formation by calculating total tube length in four randomly chosen fields from each well. F, Quantitative analysis of transwells was performed by counting crystal violet positive cells in four randomly chosen fields from each well. G, Apoptotic index analysis, (n = 5). H, Representative blots of cleaved caspase‐3, caspase‐3, BAX, Bcl‐2, SIRT1, Ac‐p53 (Lys382), p‐p53 (Thr55 and Ser46), p53 and hydroxylated HIF‐1α (Pro564) in each group. I‐L, Western blotting was used to analyse the expression of cleaved caspase‐3 (c caspase‐3), BAX, Bcl‐2 and SIRT1 in each group. M, The levels of SIRT1 mRNA in each group. N‐Q, Western blotting was used to analyse the expression of Ac‐p53 (Lys382), p‐p53 (Thr55 and Ser46) and hydroxylated HIF‐1α (Pro 564) in each group (n = 5). Error bars represent mean ± SEM. *P < .05 between indicated groups

To determine the effects of T0901317 on cell viability during H/SD + HG injury, HUVECs were incubated with different concentrations (10 nmol/L, 20 nmol/L, 50 nmol/L, 100 nmol/L and 150 nmol/L) of T0901317 for 24 hours, which was followed by H/SD + HG treatment. Our results revealed that HUVECs suffered a greater loss after H/SD + HG injury than what was observed in the H/SD group (Figure 5A). T0901317 at a concentration of 100 nmol/L efficiently rescued HUVECs from H/SD + HG injury (H/SD + HG *vs* H/SD + HG+100 nmol/L group). Thus, 100 nmol/L T0901317 was used in the subsequent study. Furthermore, the effect of SIRT1 siRNA was evaluated by Western blotting and the results are shown in Figure 5B‐C. The expression of SIRT1 notably decreased as SIRT1 siRNA was administered to HUVECs.

SIRT1 silencing decreased H/SD‐induced tube formation and migration and increased the number of apoptotic HUVECs in the HG context compared to what was observed in the H/SD + HG group. LXR agonist pre‐treatment attenuated endothelial cell apoptosis and increased tube formation and migration compared to those of the H/SD + HG group. The beneficial effects of LXR agonist treatment were largely counteracted by SIRT1 silencing; however, they were still present in the H/SD + HG+LXR + si SIRT1 group (*vs* H/SD + HG+si SIRT1 group, Figure 5D‐G).

**Figure 6:**
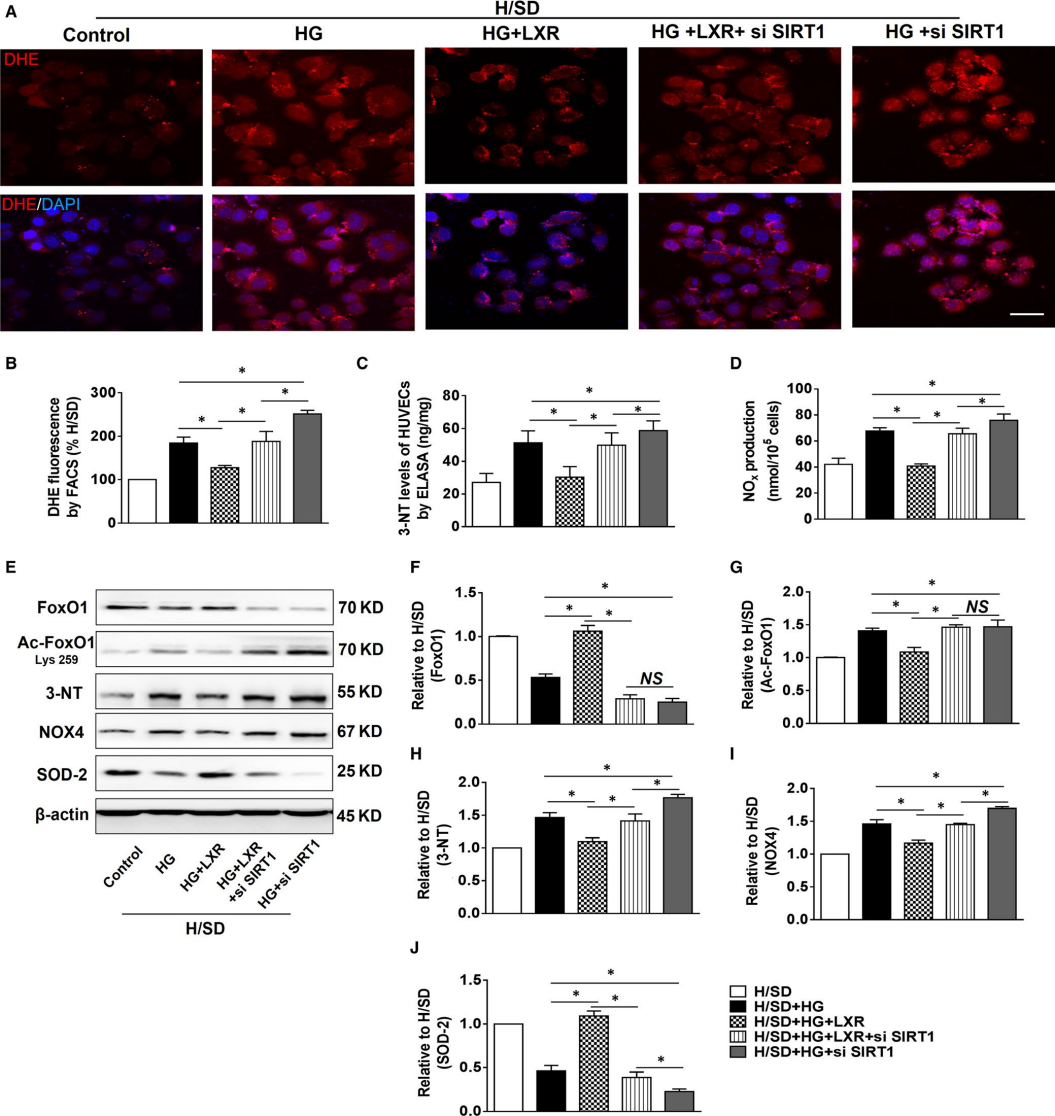
The LXR agonist T0901317 mitigated endothelial oxidative and nitrative stress in H/SD + HG injury. A, Representative images of dihydroethidium fluorescence (red) staining were used to evaluate oxidative stress. Scale bar: 30 µm. B, Intracellular ROS generation was measured by flow cytometry through the analysis of DHE fluorescence (n = 5). C, Measurement of 3‐NT by ELISA (n = 5). D, Intracellular NOx contents in different groups (n = 5). E, Representative blots of FoxO1, Ac‐FoxO1 (Lys259), NADPH oxidase (NOX4), 3‐nitrotyrosine (3‐NT) and SOD‐2. F‐J, Western blotting was used to analyse the expression of FoxO1, Ac‐FoxO1 (Lys259), 3‐NT, NOX4 and SOD‐2 in each group. (n = 5). *P < .05 between the indicated groups

Furthermore, to elucidate the underlying mechanism of these effects, we evaluated the expression of apoptosis‐ and angiogenesis‐related proteins as well as the levels of SIRT1. LXR agonist treatment down‐regulated the levels of BAX and cleaved caspase‐3 and up‐regulated Bcl‐2 compared to those of the H/SD + HG group. These LXR‐elicited trends were reversed by SIRT1 silencing; however, they were still occurred in the H/SD + HG+si SIRT1 group (Figure 5H‐K). Furthermore, we found that LXR agonist treatment up‐regulated SIRT1 at both the mRNA and protein levels under H/SD + DM conditions (Figure 5L‐M). Interestingly, LXR agonist treatment down‐regulated the level of Ac‐p53, a downstream target of SIRT1; however, this trend did not occur under SIRT1 silencing conditions. Moreover, the levels of phosphorylated‐p53 (Thr55 and Ser46) were down‐regulated by LXR agonist treatment, regardless of whether SIRT1 was silenced (Figure 6N‐P). Additionally, the level of hydroxylated hypoxia‐inducible factor‐1*α* (hydroxylated HIF‐1*α*) was still down‐regulated and the level of HIF‐1*α* was up‐regulated in the H/SD + HG+LXR + si SIRT1 group (*vs* H/SD + HG+si SIRT1 group, Figure 5Q‐R).

### 3.6 SIRT1 silencing exacerbated endothelial OS and inhibited the antioxidative effects of the LXR agonist in response to H/SD + HG

SIRT1 silencing exacerbated endothelial OS in the H/SD + HG injury group compared to that in the H/SD + HG group, as evidenced by increased mean fluorescence intensity (Figure 6A‐B) and the levels of 3‐nitrotyrosine (3‐NT) and NOx (Figure 6C‐D). LXR agonist pre‐treatment mitigated OS in HUVECs in response to H/SD + HG compared with that in the H/SD + HG group. Moreover, the LXR‐elicited effects were compromised by SIRT1 silencing (H/SD + HG+LXR *vs* H/SD + HG+LXR + si SIRT1 group); however, they were still present in the H/SD + HG+LXR + si SIRT1 group (*vs* H/SD + HG+si SIRT1 group, Figure 6A‐D).

Furthermore, we assessed OS‐related proteins to determine the mechanism by which LXR agonist treatment elicited antioxidative effects (Figure 6E). Forkhead box transcription factor O1 (FoxO1), a target of SIRT1 that is related to OS, was also involved in our investigation. LXR agonist treatment down‐regulated the level of Ac‐FoxO1 compared to that of the H/SD + HG group; however, this trend was reversed by SIRT1 silencing (Figure 6F‐G). Additionally, LXR agonist treatment decreased 3‐NT and NADPH oxidase 4 (NOX4), which is a constitutive NADPH oxidase and generates intracellular superoxide; further, LXR agonist treatment increased SOD‐2 expression compared to that of the H/SD + HG group (Figure 6H‐J). These LXR‐elicited trends were reversed by SIRT1 silencing; however, the trends were still present compared to those of the H/SD + HG+si SIRT1 group (Figure 6H‐J).

### 3.7 The isoform LXR*β* was involved in LXR agonist‐elicited SIRT1 regulation

**Figure 7:**
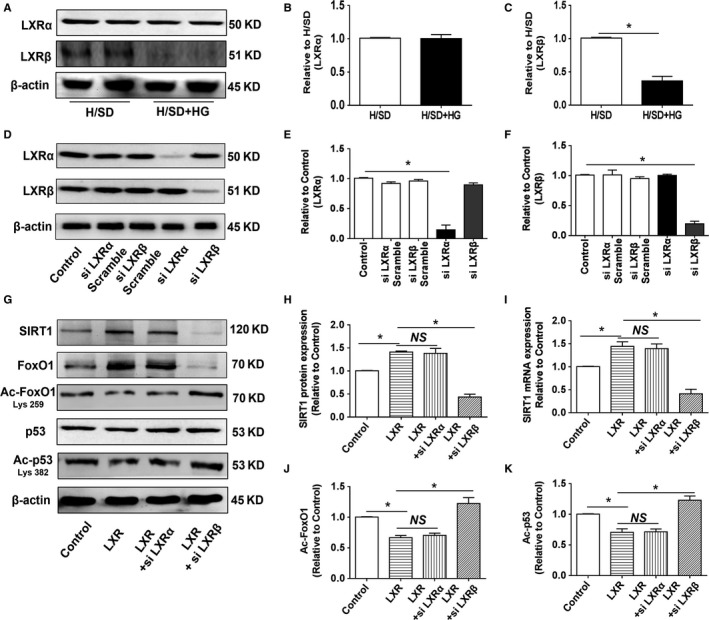
The isoform LXR*β* was involved in LXR agonist‐elicited SIRT1 regulation. A, Representative blots of LXR*α* and LXR*β*. Lane 2 and lane 4 were duplicates of lanes 1 and 3, respectively. B‐C, Western blotting was used to analyse the expression of LXR*α* and LXR*β* (n = 5). D‐F, The effects of LXR*α* and LXR*β* siRNA intervention were assessed by Western blotting (n = 3). G, Representative blots of SIRT1, FoxO1, Ac‐FoxO1, p53 and Ac‐p53 in each group. H‐I, The levels of SIRT1 mRNA and protein in response to T0901317 under LXR*α* or LXR*β* silencing conditions (n = 5). J‐K, Western blotting was used to analyse the expression of Ac‐FoxO1 and Ac‐p53 (n = 5). *P < .05 between the indicated groups

Since LXR*α* and LXR*β* are distributed in specific organs and tissues, we explored which isoform was involved in ECs. As shown in Figure 7A‐C, H/SD + HG treatment decreased LXR*β*; however, the LXR*α* level was not decreased. To investigate the effects of the two isoforms, we treated cells with LXR*α* and LXR*β* siRNAs to evaluate the response to T0901317. The expression of LXR*α* and LXR*β* decreased in HUVECs following treatment with the respective siRNAs (Figure 7D‐F). However, as shown in Figure 7G‐K, silencing of LXR*β*, not LXR*α*, counteracted LXR agonist‐elicited SIRT1 up‐regulation, as well as acetyl‐p53 and acetyl‐FoxO1 down‐regulation under basal conditions. Therefore, the isoform LXR*β*, not LXR*α*, may be involved in LXR agonist‐elicited SIRT1 regulation.

## 4 DISCUSSION

Intact and well‐functioning ECs are indispensable for angiogenesis and revascularization.^19^, ^20^ However, DM causes metabolic disorders, especially hyperglycaemia, impairing ischaemia‐induced angiogenesis and deteriorating endothelial injury in the context of PAD.^21^ Our present study investigated the effects of LXR agonist treatment on HLI + DM mice with a focus on endothelial viability and angiogenesis. For the first time, our results revealed that LXR agonist treatment markedly protected against hindlimb ischaemia in diabetic mice by attenuating endothelial apoptosis and OS, resulting in promoting of angiogenesis.

DM patients suffer from an excessive level of OS associated with increased ROS production, as well as reduced endogenous antioxidants.^22^ On the one hand, chronic hyperglycaemia induces advanced glycosylation end product (AGE) binding to the receptors of AGEs^23^ and activation of the diacylglycerol (DAG)‐protein kinase C pathway^22^; as a consequence of NOX activation, excessive ROS formation occurs and initiates a vicious cycle of OS.^24^ On the other hand, endogenous antioxidants are inevitably reduced in DM, including antioxidant enzymes and non‐enzymatic compounds, both of which could detoxify ROS directly.^25^ Undue OS accelerates endothelial senescence and exacerbates apoptosis and dysfunction, reducing ischaemia‐induced neovascularization as a result.^26^ Hence, a treatment focused on alleviating endothelial OS may be a potentially useful treatment for diabetic vascular complications.

Activated SIRT1 deacetylates and regulates its downstream targets, performing various beneficial functions in metabolic disorder‐related vascular disease.^15^ SIRT1‐induced deacetylation and activation of FoxO1 and proliferator‐activated receptor gamma coactivator 1‐alpha inhibits OS *via* inducing endogenous antioxidant enzymes such as SOD, catalase and the non‐enzymatic compound GSH,^15^, ^25^, ^27^, ^28^ as well as *via* FoxO1‐c‐Myc signalling or through direct regulation of VEGF expression.^29^, ^30^ We evaluated the gastrocnemius tissue to determine the contents of VEGF and bFGF secreted by surviving and regenerated cells (muscle cells, ECs, fibroblast cells, smooth muscle cells, etc), which were salvaged from angiogenesis. Therefore, VEGF and bFGF in the gastrocnemius were indirect evidence for angiogenesis. Additionally, SIRT1 activation promotes endothelial functions,^31^ such as migration and proliferation, which are essential for angiogenesis.^32^ Furthermore, SIRT1 also directly regulates OS and antioxidative stress, which involves the SIRT1/NOX, SIRT1/SOD and SIRT1/eNOS pathways.^28^ In contrast, SIRT1 inhibition causes OS in patients with cardiovascular disease,^33^ impaired endothelium‐dependent vasorelaxation and hypoxia‐induced angiogenesis.^34^, ^35^ We found that the LXR agonist T0901317 up‐regulated SIRT1 at both the mRNA and protein levels and further regulated the SIRT1 downstream targets FoxO1 and p53. Endothelial functions were preserved by an LXR agonist‐elicited antioxidant effect, as evidenced by decreased levels of ROS and MDA, a well‐investigated end product of lipid oxidation, as well as increased levels of the antioxidant enzymes SOD and CAT or the non‐protein low molecular weight compound GSH. Furthermore, we also found that LXR agonist treatment blocked the vicious cycle of ROS by decreasing NOX4 expression in H/SD + HG injury. Interestingly, our studies found that treatment with a 50 mg/kg dose of T0901317 reduced blood reperfusion of ischaemic hindlimb, which may be triggered by inhibiting the physiological level of oxidative stress‐induced angiogenesis.^36^, ^37^

SIRT1 deficiency largely counteracted the therapeutic effects of LXR agonist treatment. However, the effects were partly present compared to those of the SIRT1 deficiency group with HLI + DM or H/SD + HG injury. Therefore, we hypothesized that non‐SIRT1 signalling may be involved in the LXR agonist‐elicited beneficial effects, and we further explored the underlying mechanisms in vitro. Our current results demonstrated that SIRT1 silencing only blocked LXR agonist‐induced changes in acetylated‐FoxO1 and acetylated‐p53; changes were not observed in the phosphorylation of p53 at Ser46 and Thr55, which are closely related to apoptosis and OS.^38^, ^39^ In fact, either acetylation or phosphorylation only led to partial p53 activation, and both modifications were required for complete p53 activation.^40^ Hence, various LXR agonist‐induced post‐translational modifications of p53 may contribute to partly inhibiting OS and apoptosis in SIRT1 deletion conditions. Furthermore, the level of hydroxylated HIF‐1*α*, which is related to hypoxia‐induced angiogenesis by regulating the degradation of HIF‐1*α*,^41^, ^42^ was still inactivated by LXR agonist treatment when SIRT1 was silenced, which may be a compensatory mechanism for angiogenesis in SIRT1 deficiency.

Although previous studies reported a link between SIRT1 deacetylation and activation of LXR, SIRT1 deletion compromised the normal responses to the LXR agonist and eliminated the antioxidative stress and anti‐apoptotic effects of LXR agonist treatment.^11^, ^16^ Therefore, we explored whether blocking LXR could hinder LXR agonist‐elicited SIRT1 regulation. Since LXRs consist of two different and highly homologous isoforms that are distributed in specific organs and tissues,^43^ LXR*α* and LXR*β*, we explored which isoform was involved in EC. In our experience, HG exposure decreased LXR*β* but not LXR*α* expression under H/SD conditions. Furthermore, silencing LXR*β* but not LXR*α* compromised LXR agonist‐elicited SIRT1 up‐regulation at both the mRNA and protein levels. Based on the above results, we determined that the isoform LXR*β* might contribute to LXR agonist‐elicited SIRT1 regulation. Several previous reports have demonstrated that activation of LXR*α*, which is specifically distributed in the myocardium, protects against cardiovascular disease by maintaining glucose homeostasis and mitigating myocardial apoptosis and OS.^8^, ^9^ Unlike LXR*α*, LXR*β* is more ubiquitously expressed and highly related to endothelial functions. Activation of LXR*β* exerts a protective effect on ECs after HG exposure by inhibiting senescence and OS with an additional mechanism for vascular protection.^10^ In addition, LXR activation leads to LXR*β*‐ and ER*α*‐dependent processes, facilitating EC migration and preserving endothelial integrity by stimulating endothelial NO production.^44^ These results further indicated that the endothelial LXR*β* isoform may contribute to LXR agonist‐elicited beneficial effects.

In conclusion, our current study revealed a protective role of LXR agonist treatment in diabetic mice with HLI *via* promoting endothelial viability, mitigating OS and enhancing angiogenesis. Then, we found that endothelial SIRT1 played a crucial role in LXR agonist‐elicited beneficial effects. In vitro studies in HUVECs revealed that the underlying therapeutic mechanisms of LXR agonist treatment were related to SIRT1 and non‐SIRT1 signalling, and the isoform LXR*β* was involved in LXR agonist‐elicited SIRT1 regulation. Our findings further elucidate the interaction between LXR and SIRT1 and may provide positive evidence for further clinical trials to assess the potential therapeutic effect of LXR agonists in diabetic patients with vascular lesions.

## CONFLICT OF INTEREST

The authors declare that they have no conflict of interest.

## AUTHOR CONTRIBUTION

Feng Cao and Wensi Fan designed the research study; Wensi Fan, Ran Zhang, Shuang Li and Han Dong performed the experiments; JiBin Zhang and YanHua Li analysed the data; Feng Cao and Wensi Fan wrote the paper; and Zhenhua Jiang and YaBin Wang participated in the revision of the manuscript. All authors approve of the final version to be published.

## Data Availability

The data that support the findings of this study are available from the corresponding author upon reasonable request.
